# The effectiveness of digital twins in promoting precision health across the entire population: a systematic review

**DOI:** 10.1038/s41746-024-01146-0

**Published:** 2024-06-03

**Authors:** Mei-di Shen, Si-bing Chen, Xiang-dong Ding

**Affiliations:** 1 School of Nursing, Peking University, Beijing, China; 2 Department of Plastic and Reconstructive Microsurgery, China-Japan Union Hospital, Jilin University, Changchun, Jilin China

**Keywords:** Public health, Diseases, Signs and symptoms, Risk factors

## Abstract

Digital twins represent a promising technology within the domain of precision healthcare, offering significant prospects for individualized medical interventions. Existing systematic reviews, however, mainly focus on the technological dimensions of digital twins, with a limited exploration of their impact on health-related outcomes. Therefore, this systematic review aims to explore the efficacy of digital twins in improving precision healthcare at the population level. The literature search for this study encompassed PubMed, Embase, Web of Science, Cochrane Library, CINAHL, SinoMed, CNKI, and Wanfang Database to retrieve potentially relevant records. Patient health-related outcomes were synthesized employing quantitative content analysis, whereas the Joanna Briggs Institute (JBI) scales were used to evaluate the quality and potential bias inherent in each selected study. Following established inclusion and exclusion criteria, 12 studies were screened from an initial 1321 records for further analysis. These studies included patients with various conditions, including cancers, type 2 diabetes, multiple sclerosis, heart failure, qi deficiency, post-hepatectomy liver failure, and dental issues. The review coded three types of interventions: personalized health management, precision individual therapy effects, and predicting individual risk, leading to a total of 45 outcomes being measured. The collective effectiveness of these outcomes at the population level was calculated at 80% (36 out of 45). No studies exhibited unacceptable differences in quality. Overall, employing digital twins in precision health demonstrates practical advantages, warranting its expanded use to facilitate the transition from the development phase to broad application.

PROSPERO registry: CRD42024507256.

## Introduction

Precision health represents a paradigm shift from the conventional “one size fits all” medical approach, focusing on specific diagnosis, treatment, and health management by incorporating individualized factors such as omics data, clinical information, and health outcomes^1,2^. This approach significantly impacts various diseases, potentially improving overall health while reducing healthcare costs^3,4^. Within this context, digital twins emerged as a promising technology^5^, creating digital replicas of the human body through two key steps: building mappings and enabling dynamic evolution^6^. Unlike traditional data mining methods, digital twins consider individual variability, providing continuous, dynamic recommendations for clinical practice^7^. This approach has gained significant attention among researchers, highlighting its potential applications in advancing precision health.

Several systematic reviews have explored the advancement of digital twins within the healthcare sector. One rapid review^8^ identified four core functionalities of digital twins in healthcare management: safety management, information management, health management/well-being promotion, and operational control. Another systematic review^9^, through an analysis of 22 selected publications, summarized the diverse application scenarios of digital twins in healthcare, confirming their potential in continuous monitoring, personalized therapy, and hospital management. Furthermore, a quantitative review^10^ assessed 94 high-quality articles published from 2018 to 2022, revealing a primary focus on technological advancements (such as artificial intelligence and the Internet of Things) and application scenarios (including personalized, precise, and real-time healthcare solutions), thus highlighting the pivotal role of digital twins technology in the field of precision health. Another systematic review^11^, incorporating 18 framework papers or reviews, underscored the need for ongoing research into digital twins’ healthcare applications, especially during the COVID-19 pandemic. Moreover, a systematic review^12^ on the application of digital twins in cardiovascular diseases presented proof-of-concept and data-driven approaches, offering valuable insights for implementing digital twins in this specific medical area.

While the existing literature offers valuable insights into the technological aspects of digital twins in healthcare, these systematic reviews failed to thoroughly examine the actual impacts on population health. Despite the increasing interest and expanding body of research on digital twins in healthcare, the direct effects on patient health-related outcomes remain unclear. This knowledge gap highlights the need to investigate how digital twins promote and restore patient health, which is vital for advancing precision health technologies. Therefore, the objective of our systematic review is to assess the effectiveness of digital twins in improving health-related outcomes at the population level, providing a clearer understanding of their practical benefits in the context of precision health.

## Results

### Search results

**Fig. 1:**
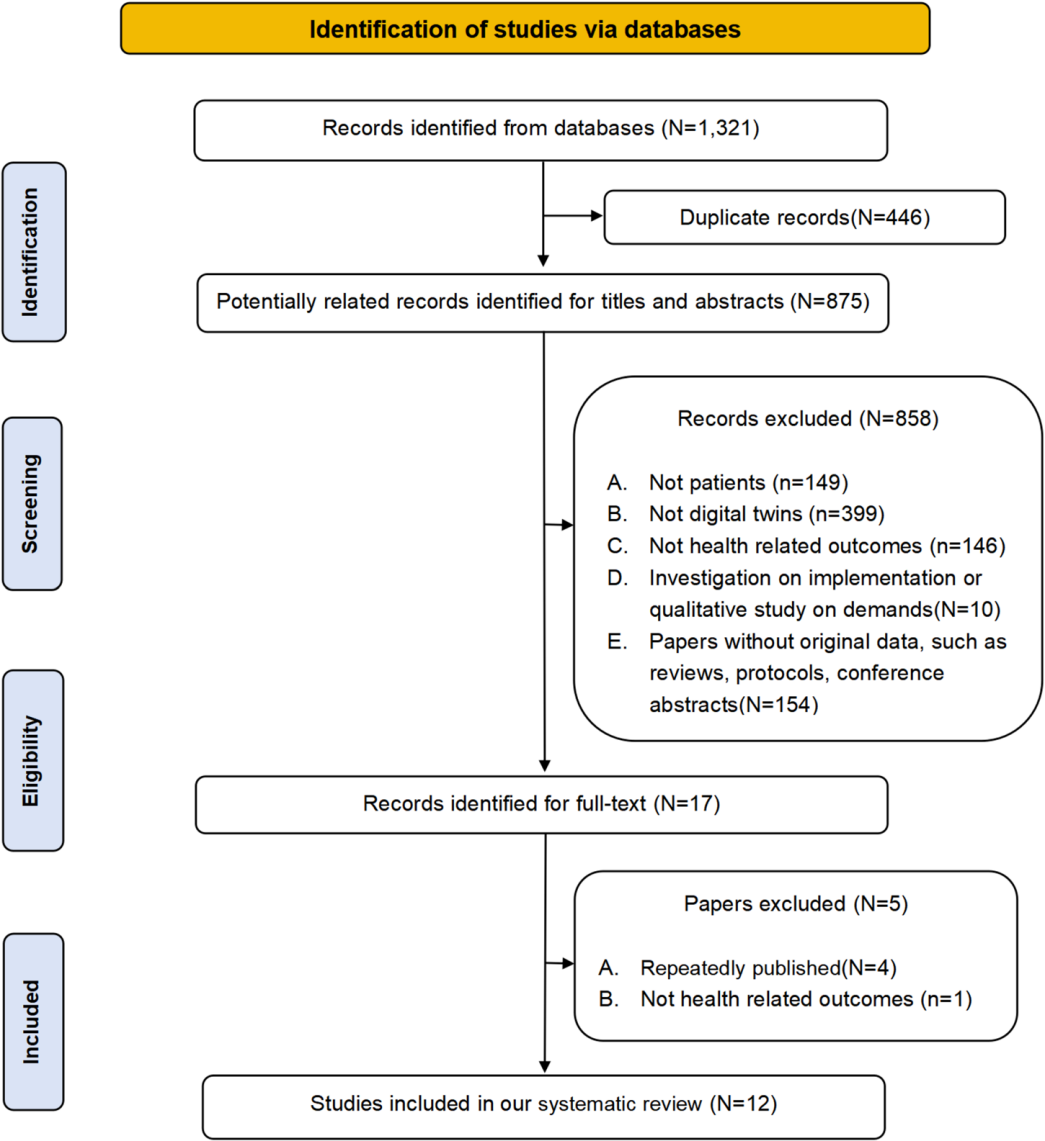
Flow chart of included studies in the systematic review.

The selection process for the systematic review is outlined in the PRISMA flow chart (Fig. 1). Initially, 1321 records were identified. Of these, 446 duplicates (446/1321, 33.76%) were removed, leaving 875 records (875/1321, 66.24%) for title and abstract screening. Applying the pre-defined inclusion and exclusion criteria led to the exclusion of 858 records (858/875, 98.06%), leaving 17 records (17/875, 1.94%) for full-text review. Further scrutiny resulted in the exclusion of one study (1/17, 5.88%) lacking health-related outcomes and four studies (4/17, 23.53%) with overlapping data. Ultimately, 12 (12/17, 70.59%) original studies^13–24^ were included in the systematic review. Supplementary Table 1 provides a summary of the reasons for exclusion at the full-text reading phase.

### Study characteristics

The studies included in this systematic review were published between 2021 (2/12, 16.67%)^23,24^ and 2023 (8/12, 66.67%)^13–20^. Originating from diverse regions, 4/12 studies (33.33%) were from Asia^13,14,21,24^, 5/12 (41.67%) from America^15,17,19,20,22^, and 3/12 (25.00%) from Europe^16,18,23^. The review encompassed various study designs, including randomized controlled trials (1/12, 8.33%)^14^, quasi-experiments (6/12, 50.00%)^13,15,16,18,19,21^, and cohort studies (5/12, 41.67%)^17,20,22–24^. The sample sizes ranged from 15^13^ to 3500 patients^19^. Five studies assessed the impact of digital twins on virtual patients^15,16,18–20^, while seven examined their effect on real-world patients^13,14,17,21–24^. These patients included had various diseases, including cancer (4/12, 33.33%)^15,16,19,22^, type 2 diabetes (2/12, 16.66%)^13,14^, multiple sclerosis (2/12, 16.66%)^17,18^, qi deficiency (1/12, 8.33%)^21^, heart failure (1/12, 8.33%)^20^, post-hepatectomy liver failure (1/12, 8.33%)^23^, and dental issues (1/12, 8.33%)^24^. This review coded interventions into three types: personalized health management (3/12, 25.00%)^13,14,21^, precision individual therapy effects (3/12, 25.00%)^15,16,18–20,22^, and predicting individual risk (3/12, 25.00%)^17,23,24^, with a total of 45 measured outcomes. Characteristics of the included studies are detailed in Table 1.

**Table 1:** Characteristics of the included studies

Study ID	Year	Country	Study Type	Sample Size	Population	Intervention	Controls	Measurements	Appraisal
Thamotharan et al.	2023	India	Quasi-experiment study^a^	15	Elderly with type 2 diabetes	Individualized insulin infusion	NA	TIR; Percentage of improvement in hypo; Percentage of improvement in hyper	The study lacked any control group, and it only presented baseline data without follow-up.
Joshi et al.	2023	India	Randomized controlled trial	319	Patients with type 2 diabetes	Personalized meal plan	Standard care	Weight; BMI; WC; HbA1C; HOMA2-IR; ALT; AST; GGT; NAFLD-LFS; NAFLD-NFS; FLI; FSI; FIB4; APRI; CV; MAGE; hs-CRP; WBC; ESR; Ferritin	The study only provided baseline data without reporting the comparability of the baselines between groups. Additionally, there was no blinding method implemented for the subjects and the blinding method for the intervener was merely described as “recruiting the subjects”.
Chaudhuri et al.	2023	USA	Quasi-experiment study^a^	100 virtual patients by 3 sample	Patients with high-grade gliomas	Patient-specific radiotherapy regimens	NA	Tumor time to progression; Radiation dose	This study lacked control groups and inter-group comparisons at baseline. Furthermore, the study lacked any follow-up.
Bahrami et al.	2023	Switzerland	Quasi-experiment study^a^	3000 virtual patients by 20 sample	Cancer patients with pain	Individualized transdermal fentanyl therapy	NA	Average pain intensity; Median time without pain	The study lacked control groups, comparisons, and follow-up.
Cen et al.	2023	USA	Cohort study	519	Patients with multiple sclerosis	Disease-specific brain atrophy	Normal aging controls	Onset age	The study utilized data from five cohorts and included a five-year follow-up period. However, it did not provide information on the number of lost visits or the treatment measures.
Maleki et al.	2023	Italy	Quasi-experiment study^b^	3000 virtual patients by 2132 sample	Patients with multiple sclerosis	Ocrelizumab	Placebo	ARR; Lymphocytopenia adverse events	This study did not report the baseline or any follow-up measurements.
Susilo et al.	2023	USA	Quasi-experiment study^a^	3500 virtual patients by 140 sample	Patients with non-Hodgkin’s lymphoma	Different exposure and dose of mosunetuzumab	NA	Tumor size	The study did not include a control group or perform a baseline comparison. It focused solely on evaluating the tumor size as an outcome and did not provide any follow-up data.
Thangaraj et al.	2023	USA	Cohort study	2173 virtual patients by 6890 sample	Heart failure patients with preserved ejection fraction	Spironolactone	Placebo	5-year cardiovascular composite outcome	The study utilized data from two cohorts and included a five-year follow-up period. However, it did not provide information on the number of lost visits or the treatment measures.
Jiang et al.	2022	China	Quasi-experiment study^b^	100	Patients with Qi deficiency	TCM physical health management	Western medicine nursing	HR; BMI; BP; Main TCM symptoms; Secondary TCM symptoms; Total score of TCM syndromes; MLHFQ	The study lacked a clear explanation of the causal relationship and did not conduct any follow-up.
Tardini et al.	2022	USA	Cohort study	134	Patients with oropharyngeal squamous carcinomas	Optimal treatment selection	Doctor decision-making	Survival rate; Dysphagia rate	The study did not report the number of lost visits or provide information on the treatment measures.
Golse et al.	2021	France	Cohort study	47	Patients with major hepatectomy	Post-hepatectomy liver failure	Without post-hepatectomy liver failure	PPV; PCG	The study reported a follow-up period of 90 days but failed to disclose details regarding follow-up losses or treatment measures.
Cho et al.	2021	Korea	Cohort study	50	Adult female patients with dental problems	Immediate orthodontic treatment	Finished the orthodontic treatment	Maxillary central incisors; Forehead inclination	The study failed to account for or regulate confounding factors, and it lacked any mention of follow-up, lost follow-up information, or treatment measures.

*NA* Not applicable, *TIR* Time in range, *BMI* Body mass index, *WC* Waist circumference, *HbA1C* Hemoglobin A1c, *HOMA2-IR* Homeostatic model assessment 2 of insulin resistance, *ALT* Alanine aminotransferase, *AST* Aspartate aminotransferase, *GGT* Gamma-glutamyl transferase, *NAFLD-LFS* Nonalcoholic fatty liver disease liver fat score, *NAFLD-NFS* Nonalcoholic fatty liver disease fibrosis score, *FLI* Fatty liver index, *FSI* Framingham Steatosis Index, *FIB4* Fibrosis-4 score, *APRI* AST to Platelet Ratio Index, *CV* Coefficient of variation, *MAGE* Mean amplitude of glycemic excursion, *hs-CRP* high-sensitivity C-reactive protein, *WBC* White blood cell, *ESR* Erythrocyte sedimentation rate, *ARR* Annualized Relapse Rate, *HR* Heart rate, *BP* Blood pressure, *TCM* Traditional Chinese medicine, *MLHFQ* Minnesota Heart Failure Quality of Life Scale, *PPV* High postoperative portal vein pressure, *PCG* Portocaval gradient.

^a^Self-control study.

^b^Non-randomized controlled trial.

### Risk of bias assessment

**Fig. 2:**
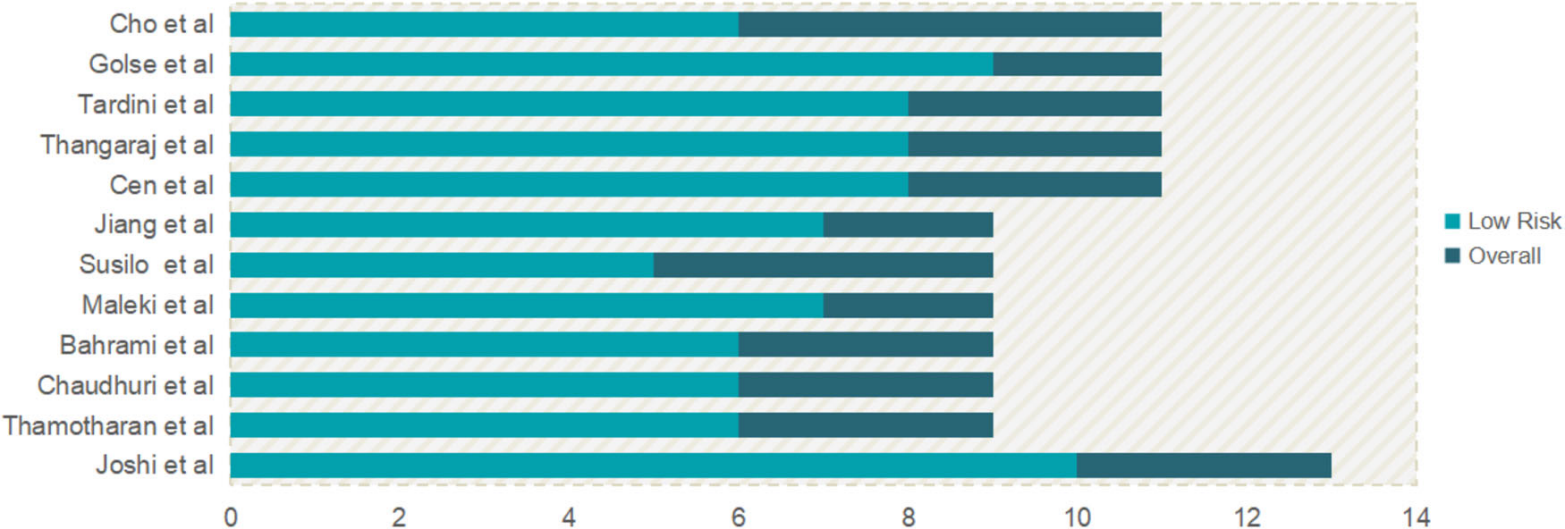
The summary of bias risk via the Joanna Briggs Institute assessment tools.

The risk of bias for the studies included in this review is summarized in Fig. 2. In the single RCT^14^ assessed, 10 out of 13 items received positive responses. Limitations were observed due to incomplete reporting of baseline characteristics and issues with blinding. Among the six quasi-experimental studies evaluated, five (83.33%)^13,15,16,18,21^ achieved at least six positive responses, indicating an acceptable quality, while one study (16.67%)^19^ fell slightly below this threshold with five positive responses. The primary challenges in these quasi-experimental studies were due to the lack of control groups, inadequate baseline comparisons, and limited follow-up reporting. Four out of five (80.00%)^17,20,22,23^ of the cohort studies met or exceeded the criterion with at least eight positive responses, demonstrating their acceptable quality. However, one study (20.00%)^24^ had a lower score due to incomplete data regarding loss to follow-up and the specifics of the interventions applied. Table 1 elaborates on the specific reasons for these assessments. Despite these concerns, the overall quality of the included studies is considered a generally acceptable risk of bias.

### The impact of digital twins on health-related outcomes among patients

**Fig. 3:**
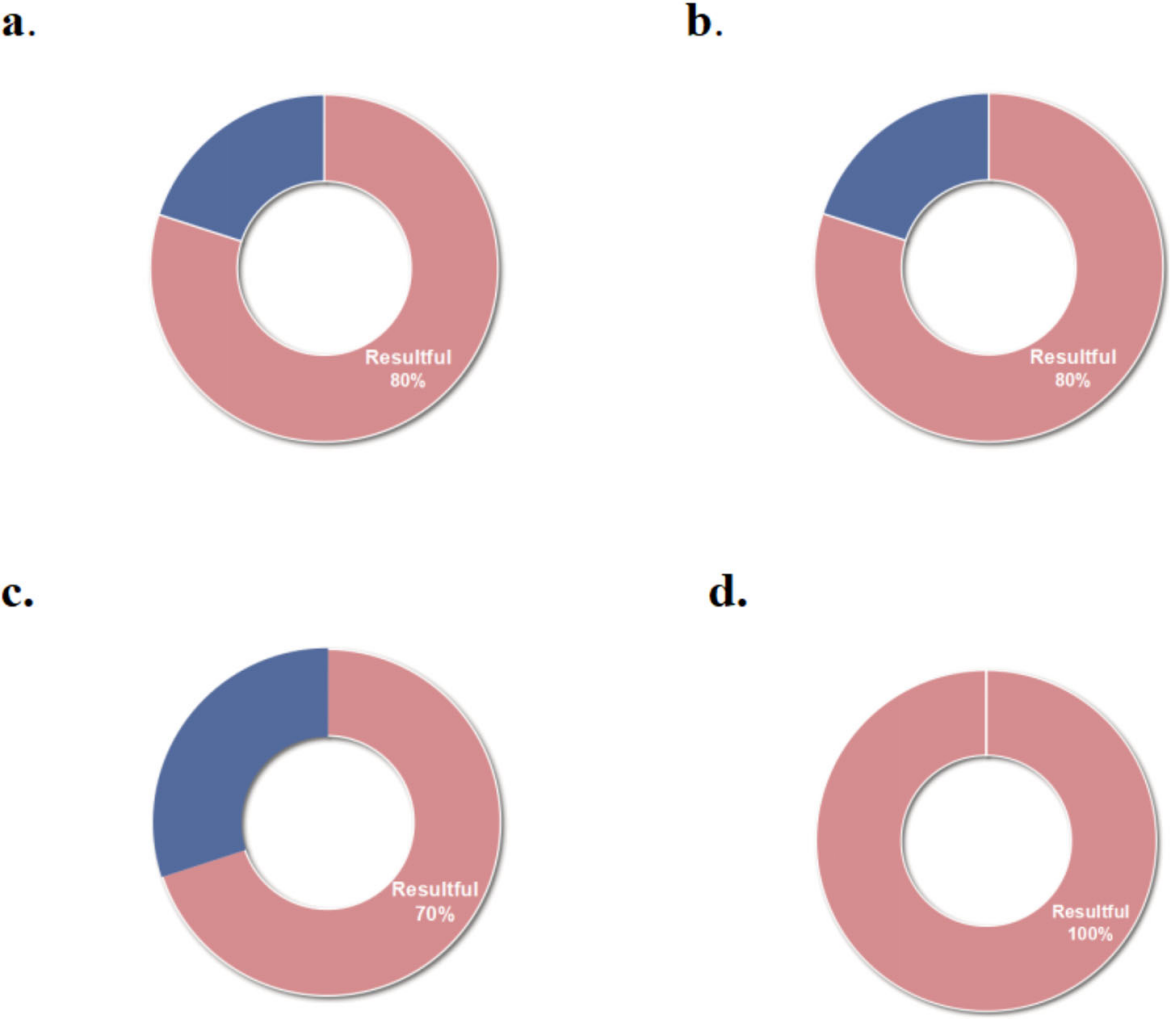
The effectiveness of digital twins. **a** The overall effectiveness of digital twins; **b** The effectiveness of personalized health management driven by digital twins; **c** The effectiveness of precision individualized therapy effects driven by digital twins; **d** The effectiveness of prediction of individual risk driven by digital twins.

This review includes 12 studies that collectively assessed 45 outcomes, achieving an overall effectiveness rate of 80% (36 out of 45 outcomes), as depicted in Fig. 3a. The digital twins analyzed were coded into three functional categories: personalized health management, precision individual therapy effects, and predicting individual risks. A comprehensive analysis of the effectiveness of digital twins across these categories is provided, detailing the impact and outcomes associated with each function.

### The effectiveness of digital twins in personalized health management

In this review, three studies^13,14,21^ employing digital twins for personalized health management reported an effectiveness of 80% (24 out of 30 outcomes), as shown in Fig. 3b. A self-control study^13^ involving 15 elderly patients with diabetes, used virtual patient representations based on health information to guide individualized insulin infusion. Over 14 days, this approach improved the time in range (TIR) from 3–75% to 86–97%, decreased hypoglycemia duration from 0–22% to 0–9%, and reduced hyperglycemia time from 0–98% to 0–12%. A 1-year randomized controlled trial^14^ with 319 type 2 diabetes patients, implemented personalized digital twins interventions based on nutrition, activity, and sleep. This trial demonstrated significant improvements in Hemoglobin A1c (HbA1C), Homeostatic Model Assessment 2 of Insulin Resistance (HOMA2-IR), Nonalcoholic Fatty Liver Disease Liver Fat Score (NAFLD-LFS), and Nonalcoholic Fatty Liver Disease Fibrosis Score (NAFLD-NFS), and other primary outcomes (all, *P* < 0.001; Table 2). However, no significant changes were observed in weight, Alanine Aminotransferase (ALT), Fibrosis-4 Score (FIB4), and AST to Platelet Ratio Index (APRI) (all, *P* > 0.05). A non-randomized controlled trial^21^ introduced a digital twin-based Traditional Chinese Medicine (TCM) health management platform for patients with qi deficiency. It was found to significantly improve blood pressure, main and secondary TCM symptoms, total TCM symptom scores, and quality of life (all, *P* < 0.05). Nonetheless, no significant improvements were observed in heart rate and BMI (all, *P* > 0.05; Table 2).

**Table 2:** The effectiveness of digital twins in precision health

Study ID	Patient Characteristics	Intervention Function Characteristics	Measurements	Results	Effectiveness	Adverse Effects
Thamotharan et al.	Type 2 diabetes	Personalized health management	TIR	(3%-75%) → (86%-97%)^a^	Y	NR
			Percentage of improvement in hypo	(0%-22%) → (0%-9%)^a^	Y	
			Percentage of improvement in hyper	(0%-98%) → (0%-12%)^a^	Y	
Joshi et al.			Weight	*p* = 0.688	N	NR
			BMI	*p* < 0.001	Y	
			WC	*p* < 0.001	Y	
			HbA1C	*p* < 0.001	Y	
			HOMA2-IR	*p* < 0.001	Y	
			ALT	*p* = 0.821	N	
			AST	*p* < 0.001	Y	
			GGT	*p* < 0.001	Y	
			NAFLD-LFS	*p* < 0.001	Y	
			NAFLD-NFS	*p* < 0.001	Y	
			FLI	*p* < 0.001	Y	
			FSI	*p* < 0.001	Y	
			FIB4	*p* = 0.233	N	
			APRI	*p* = 0.526	N	
			CV	*p* < 0.001	Y	
			MAGE	*p* < 0.001	Y	
			hs-CRP	*p* < 0.001	Y	
			WBC	*p* < 0.001	Y	
			ESR	*p* < 0.001	Y	
			Ferritin	*p* = 0.002	Y	
Jiang et al.	Qi deficiency		HR	*p* = 0.333	N	NR
			BMI	*p* = 0.637	N	
			BP	*p* < 0.001	Y	
			Main TCM symptoms	*p* < 0.05	Y	
			Secondary TCM symptom	*p* < 0.05	Y	
			Total score of TCM syndromes	*p* = 0.001	Y	
			MLHFQ	*p* = 0.001	Y	
Chaudhuri et al.	High-grade gliomas	Precision individualized therapy effects	Tumor time to progression	+ 6 days^a^	Y	NR
			Radiation dose	- 16.7%^a^	Y	
Bahrami et al.	Cancer pain		Average pain intensity	- 16%^a^	Y	NR
			Median time without pain	+ 23 hours^a^	Y	
Susilo et al.	Non-Hodgkin’s lymphoma		Tumor size	- 50%^a^	Y	NR
Thangaraj et al.	Heart failure		5-year cardiovascular composite outcome	0.85 (0.69, 1.04)	N	NR
Tardini et al.	Oropharyngeal squamous carcinomas		Survival rate	+3.73(-0.75, 8.96)	N	Risk of Dysphagia
			Dysphagia rate	+0.75 (-4.48, 6.72)	N	
Maleki et al.	Multiple sclerosis		ARR	0.191 (0.143, 0.239)	Y	NR
			Lymphocytopenia adverse events	19.9% VS 83.73%^a^	Y	
Cen et al.		Prediction of individual risk	Onset age	*p* < 0.01	Y	NR
Golse et al.	Major hepatectomy		PPV	*r* = 0.75, *p* < 0.0001	Y	NR
			PCG	*r* = 0.80, *p* < 0.0001	Y	
Cho et al.	Dental problems		Maxillary central incisors	*p* = 0.04	Y	NR
			Forehead inclination	*p* = 0.02	Y	

*NR* Not report, *Y* Resultful, *N* Resultless, *TIR* Time in range, *BMI* Body mass index, *WC* Waist circumference, *HbA1C* Hemoglobin A1c, *HOMA2-IR*:Homeostatic model assessment 2 of insulin resistance, *ALT* Alanine aminotransferase, *AST* Aspartate aminotransferase, *GGT* Gamma-glutamyl transferase, *NAFLD-LFS* Nonalcoholic fatty liver disease liver fat score, *NAFLD-NFS* Nonalcoholic fatty liver disease fibrosis score, *FLI* Fatty liver index, *FSI* Framingham Steatosis Index, *FIB4* Fibrosis-4 score, *APRI* AST to Platelet Ratio Index, *CV* Coefficient of variation, *MAGE* Mean amplitude of glycemic excursion, *hs-CRP* high-sensitivity C-reactive protein, *WBC* White blood cell, *ESR* Erythrocyte sedimentation rate, *HR* Heart rate, *BP* Blood pressure, *TCM* Traditional Chinese medicine, *MLHFQ* Minnesota Heart Failure Quality of Life Scale, *ARR* Annualized Relapse Rate, *PPV* High postoperative portal vein pressure, *PCG* Portocaval gradient

^a^Authors self-reported a significant changes.

### The effectiveness of digital twins in precision individual therapy effects

Six studies^15,16,18–20,22^ focused on the precision of individual therapy effects using digital twins, demonstrating a 70% effectiveness rate (7 out of 10 outcomes), as detailed in Fig. 3c. In a self-control study^15^, a data-driven approach was employed to create digital twins, generating 100 virtual patients to predict the potential tumor biology outcomes of radiotherapy regimens with varying contents and doses. This study showed that personalized radiotherapy plans derived from digital twins could extend the median tumor progression time by approximately six days and reduce radiation doses by 16.7%. Bahrami et al.^16^ created 3000 virtual patients experiencing cancer pain to administer precision dosing of fentanyl transdermal patch therapy. The intervention led to a 16% decrease in average pain intensity and an additional median pain-free duration of 23 hours, extending from 72 hours in cancer patients. Another quasi-experimental study^18^ created 3000 virtual patients with multiple sclerosis to assess the impact of Ocrelizumab. Findings indicated Ocrelizumab can resulted in a reduction in relapses (0.191 [0.143, 0.239]) and lymphopenic adverse events (83.73% *vs*. 19.9%) compared to a placebo. American researchers^19^ developed a quantitative systems pharmacology model using digital twins to identify the optimal dosing for aggressive non-Hodgkin lymphoma patients. This approach resulted in at least a 50% tumor size reduction by day 42 among 3500 virtual patients. A cohort study^20^ assessed the 5-year composite cardiovascular outcomes in 2173 virtual patients who were treated with spironolactone or left untreated and indicated no statistically significant inter-group differences (0.85, [0.69–1.04]). Tardini et al.^22^ employed digital twins to optimize multi-step treatment for oropharyngeal squamous cell carcinoma in 134 patients. The optimized treatment selection through digital twins predicted increased survival rates by 3.73 (−0.75, 8.96) and dysphagia rates by 0.75 (−4.48, 6.72) compared to clinician decisions, with no statistical significance.

### The effectiveness of digital twins in predicting individual risk

Three studies^17,23,24^ employing digital twins to predict individual patient risks demonstrated a 100% effectiveness rate (5 out of 5 outcomes), as shown in Fig. 3d. A cohort study^17^ used digital twins to forecast the onset age for disease-specific brain atrophy in patients with multiple sclerosis. Findings indicated that the onset of progressive brain tissue loss, on average, preceded clinical symptoms by 5-6 years among the 519 patients (*P* < 0.01). Another study^23^ focused on predicting postoperative liver failure in 47 patients undergoing major hepatectomy through mathematical models of blood circulation. The study highlighted that elevated Postoperative Portal Vein pressure (PPV) and Portocaval Gradient (PCG) values above 17.5 mmHg and 13.5 mmHg, respectively, correlated with the measured values (all, *P* < 0.0001; Table 2). These indicators were effective in predicting post-hepatectomy liver failure, accurately identifying three out of four patients who experienced this complication. Cho et al.^24^ created digital twins for 50 adult female patients using facial scans and cone-beam computed tomography images to evaluate the anteroposterior position of the maxillary central incisors and forehead inclination. The analysis demonstrated significant differences in the position of the maxillary central incisors (*P* = 0.04) and forehead inclination (*P* = 0.02) between the two groups.

## Discussion

This systematic review outlines the effectiveness of digital twins in improving health-related outcomes across various diseases, including cancers, type 2 diabetes, multiple sclerosis, qi deficiency, heart failure, post-hepatectomy liver failure, and dental issues, at the population level. Distinct from prior reviews that focused on the technological dimensions of digital twins, our analysis shows the practical applications of digital twins in healthcare. The applications have been categorized into three main areas: personalized health management, precision individual therapy effects, and predicting individual risks, encompassing a total of 45 outcomes. An overall effectiveness of 80% was observed across these outcomes. This review offers valuable insights into the application of digital twins in precision health and supports the transition of digital twins from construction to population-wide implementation.

Digital twins play a crucial role in achieving precision health^25^. They serve as virtual models of human organs, tissues, cells, or microenvironments, dynamically updating based on real-time data to offer feedback for interventions on their real counterparts^26,27^. Digital twins can solve complex problems in personalized health management^28,29^ and enable comprehensive, proactive, and precise healthcare^30^. In the studies reviewed, researchers implemented digital twins by creating virtual patients based on personal health data and using simulations to generate personalized recommendations and predictions. It is worth noting that while certain indicators have not experienced significant improvement in personalized health management for patients with type 2 diabetes and Qi deficiency, it does not undermine the effectiveness of digital twins. Firstly, these studies have demonstrated significant improvements in primary outcome measures. Secondly, improving health-related outcomes in chronic diseases is an ongoing, complex process heavily influenced by changes in health behaviors^31,32^. While digital twins can provide personalized health guidance based on individual health data, their impact on actual behaviors warrants further investigation.

The dual nature of medications, providing benefits yet potentially leading to severe clinical outcomes like morbidity or mortality, must be carefully considered. The impact of therapy is subject to various factors, including the drug attributes and the specific disease characteristics^33^. Achieving accurate medication administration remains a significant challenge for healthcare providers^34^, underscoring the need for innovative methodologies like computational precise drug delivery^35,36^, a example highlighted in our review of digital twins. Regarding the prediction of individual therapy effects for conditions such as cancer, multiple sclerosis, and heart failure, six studies within this review have reported partly significant improvements in patient health-related outcomes. These advancements facilitate the tailored selection and dosing of therapy, underscoring the ability of digital twins to optimize patient-specific treatment plans effectively.

Furthermore, digital twins can enhance clinical understanding and personalize disease risk prediction^37^. It enables a quantitative understanding and prediction of individuals by continuously predicting and evaluating patient data in a virtual environment^38^. In patients with multiple sclerosis, digital twins have facilitated predictions regarding the onset of disease-specific brain atrophy, allowing for early intervention strategies. Similarly, digital twins assessed the risk of liver failure after liver resection, aiding healthcare professionals in making timely decisions. Moreover, the application of digital twins in the three-dimensional analysis of patients with dental problems has demonstrated highly effective clinical significance, underscoring its potential across various medical specialties. In summary, the adoption of digital twins has significantly contributed to advancing precision health and restoring patient well-being by creating virtual patients based on personal health data and using simulations to generate personalized recommendations and predictions.

Recent studies have introduced various digital twin systems, covering areas such as hospital management^8^, remote monitoring^9^, and diagnosing and treating various conditions^39,40^. Nevertheless, these systems were not included in this review due to the lack of detailed descriptions at the population health level, which constrains the broader application of this emerging technology. Our analysis underscores the reported effectiveness of digital twins, providing unique opportunities for dynamic prevention and precise intervention across different diseases. Multiple research methodologies and outcome measures poses a challenge for quantitative publication detection. This systematic review employed a comprehensive retrieval strategy across various databases for screening articles on the effectiveness of digital twins, to reduce the omission of negative results. And four repeated publications were excluded based on authors, affiliation, population, and other criteria to mitigate the bias of overestimating the digital twins effect due to repeated publication.

However, there are still limitations. Firstly, the limited published research on digital twins’ application at the population level hinders the ability to perform a quantitative meta-analysis, possibly limiting our findings’ interpretability. We encourage reporting additional high-quality randomized controlled trials on the applicability of digital twins to facilitate quantitative analysis of their effectiveness in precision health at the population level. Secondly, this review assessed the effectiveness of digital twins primarily through statistical significance (*P*-value or 95% confidence interval). However, there are four quasi-experimental studies did not report statistical significance. One of the limitations of this study is the use of significant changes in author self-reports as a criterion in these four quasi-experimental studies for identifying effectiveness. In clinical practice, the author’s self-reported clinical significance can also provide the effectiveness of digital twins. Thirdly, by focusing solely on studies published in Chinese and English, this review may have omitted relevant research available in other languages, potentially limiting the scope of the analyzed literature. Lastly, our review primarily emphasized reporting statistical differences between groups. Future work should incorporate more application feedback from real patients to expose digital twins to the nuances of actual patient populations.

The application of digital twins is currently limited and primarily focused on precision health for individual patients. Expanding digital twins’ application from individual to group precision health is recommended to signify a more extensive integration in healthcare settings. This expansion involves sharing real-time data and integrating medical information across diverse medical institutions within a region, signifying the development of group precision health. Investigating both personalized medical care and collective health management has significant implications for improving medical diagnosis and treatment approaches, predicting disease risks, optimizing health management strategies, and reducing societal healthcare costs^41^.

Digital twins intervention encompasses various aspects such as health management, decision-making, and prediction, among others^9^. It represents a technological and conceptual innovation in traditional population health intervention. However, the current content design of the digital twins intervention is insufficient and suggests that it should be improved by incorporating more effective content strategies tailored to the characteristics of the target population. Findings from this study indicate that interventions did not differ significantly in our study is from digital twins driven by personalized health management, which means that compared with the other two function-driven digital twins, personalized health management needs to receive more attention to enhance its effect in population-level. For example, within the sphere of chronic disease management, integrating effective behavioral change strategies into digital twins is advisable to positively influence health-related indicators, such as weight and BMI. The effectiveness of such digital behavior change strategies has been reported in previous studies^42,43^. The consensus among researchers on the importance of combining effective content strategies with digital intervention technologies underscores the potential for this approach to improve patient health-related outcomes significantly.

The applications of digital twins in precision health are mainly focused on model establishment and prediction description, with limited implementation in multi-center settings. A more robust and detailed data foundation is recommended to improve clinical decision-making and reduce the likelihood of imprecise treatments. This requires continuous updating and capturing of dynamic information by digital twins in the future, as well as the improvement of the data platform that facilitates mapping, interaction, and iterative optimization. Integrating digital twins effectively into clinical workflows can support clinical interventions, assist physicians in making informed decisions, and increase the standard of patient care^6^.

The accessibility of health data is a significant challenge for the clinical implementation of digital twins. Although the internet and information technology have significantly enhanced health data availability, health data, including information systems and electronic health records, remain heterogeneous and are difficult to share^44^. Health data often contains confidential patient information, as well as unreliable information, posing challenges for implementing digital twins in healthcare settings. The primary technology utilized in digital twins, artificial intelligence algorithms, demands high-performance hardware devices and software platforms for data analysis^45^, necessitating healthcare organizations to allocate increased investment and budget for computing infrastructure supporting digital twins’ application. Therefore, future research should be focused on the technical aspects of digital twins to resolve these challenges. The automated processing of health data using a large language model and the rapid conversion of complex natural language texts into comprehensive knowledge texts are encouraged. The development of high-performance computing technology is essential for cost-effective computing requirements, which can facilitate the application of digital twins in clinical practice^46^.

Overall, this systematic review offers a comprehensive overview of digital twins in precision health, examining their impact at the population level. The findings indicate a significant overall effectiveness rate of 80% for the measured outcomes, highlighting digital twins’ pivotal role in advancing precision health. Future research should broaden the application of digital twins across various populations, integrate proven content strategies, and implement these approaches in various healthcare settings. Such efforts will maximize the benefits of digital technologies in healthcare, promoting more precise and efficacious strategies, thereby elevating patient outcomes and improving overall healthcare experiences. While digital twins offer great promise for precision health, their broad adoption and practical implementation are still in the early stages. Development, and application are essential to unlock the full potential of digital twins in revolutionizing healthcare delivery.

## Methods

This systematic review was performed following the Preferred Reporting Items for Systematic Reviews and Meta-Analysis (PRISMA) guidelines^47^. The protocol for this systematic review was prospectively registered on PROSPERO, which can be accessed *via* the following link: https://www.crd.york.ac.uk/prospero/display_record.php?ID=CRD42024507256. The registered protocol underwent an update, which included polishing the title of the article, modifying the limitation of the control group and language in the inclusion/exclusion criteria, and refining the process of data synthesis and analysis to enhance that clarity and readability of this systematic review. These modifications were updated in the revision notes section of the PROSPERO.

### Literature search strategy

Literature searches were conducted in PubMed, Embase, Web of Science, Cochrane Library, CINAHL, SinoMed, CNKI, and Wanfang Database, covering publications up to December 24, 2023. A comprehensive search strategy was developed using a combination of Medical Subject Headings terms and free-text terms, as detailed in Supplementary Table 2. Furthermore, reference lists of articles and reviews meeting the inclusion criteria were reviewed for additional relevant studies.

### Inclusion and exclusion criteria

The inclusion criteria for this systematic review included: 1) Population: Patients diagnosed with any diseases or symptoms; 2) Intervention: Any interventions involving digital twins; 3) Controls: Non-digital twin groups, such as standard care or conventional therapy, as well as no control group; 4) Outcomes: Health-related outcomes as the primary outcomes of interest; 5) Study design: All study designs that measured patient health-related outcomes after digital twins were included, including intervention studies and predictive cohort studies.

Initially, duplicates were removed. Exclusion criteria included: 1) Papers lacking original data, such as reviews, protocols, and conference abstracts; 2) Studies not in English or Chinese; 3) Surveys focusing on implementation and qualitative studies related to requirements. In cases of data duplication, the most comprehensive data report was included.

### Study selection and Data extraction

Following the automatic removal of duplicates, two independent reviewers (MD.SHEN and SB.CHEN) conducted initial screenings of titles and abstracts against the predefined inclusion and exclusion criteria to identify potentially relevant studies. Afterward, the same reviewers examined the full texts of these shortlisted articles to confirm their suitability for inclusion. This process also involved checking the reference lists of these articles for any additional studies that might meet the criteria. Data from the included studies were systematically extracted using a pre-designed extraction form. Recorded information included the first author’s name, publication year, country of origin, type of study, sample size, study population, intervention, controls, measurements, and an appraisal of each study. Disagreements between the reviewers were resolved by consultation with a third senior reviewer (XD.DING), ensuring consensus.

### Quality appraisal

The Joanna Briggs Institute (JBI) scales^48^ were used to assess the quality and potential bias of each study included in the review, employing specific tools tailored to the type of study under evaluation. These tools feature response options of “yes,” “no,” “unclear,” or “not applicable” for each assessment item. For randomized controlled trials (RCTs), the JBI scale includes 13 items, with answering “yes” to at least six items indicating a high-quality study. Quasi-experimental studies were evaluated using a nine-item checklist, where five or more positive responses qualify the research as high quality. Cohort studies underwent evaluation through an 11-item checklist, with six or more affirmative responses indicating high quality. The assessment was independently carried out by two reviewers (MD.SHEN and SB.CHEN), and any disagreements were resolved through consultation with a third senior reviewer (XD.DING), ensuring the integrity and accuracy of the quality assessment.

### Data synthesis and analysis

Given the heterogeneity in type of study and outcome measures, a meta-analysis was deemed unfeasible. Instead, a quantitative content analysis was employed to analyze all the selected studies^49,50^. Key information was extracted using a pre-designed standardized form, including the first author’s name, patient characteristics, intervention functional characteristics, measurements, results, effectiveness, and adverse events. Two reviewers (MD.SHEN and SB.CHEN) independently coded digital twin technology into three categories for descriptive analysis: personalized health management, precision individual therapy effects, and predicting individual risk, based on its functional characteristics. The Kappa statistic was applied to evaluate the inter-rater reliability during the coding process, yielding a value of 0.871, which signifies good agreement between the researchers^51,52^. The assessment of digital twins effectiveness was based on statistical significance (*P*-value or 95% confidence interval). Outcomes with statistical significance were labeled as “resultful,” whereas those lacking statistical significance were deemed “resultless.” For quasi-experimental studies, significant changes in the authors’ self-reports were used to determine the effectiveness in the absence of reporting of statistical significance. The proportion of effectiveness was calculated as the number of “resultful” indicators divided by the total number of outcomes within each category.

### Supplementary information

Supplementary File

## Data Availability

Data sharing is not applicable to this article as no datasets were generated or analyzed during the current study.

## Code availability

Code sharing is not applicable to this article as no codes were generated or analyzed during the current study.
