# Supplementary material for: The effectiveness of digital twins in promoting precision health across the entire population: a systematic review
Source: NPJ Digit Med. 2024 Jun 3;7:145. doi: 10.1038/s41746-024-01146-0 (PMC11148028; doi:10.1038/s41746-024-01146-0)
Supplement: Supplementary file 1 — Supplementary File [file 41746_2024_1146_MOESM1_ESM.pdf]

Supplementary Table 1. Papers excluded for full-text and the reasons.

	Title	Reason
No.1	Assessing over-diagnosis of fecal immunological test screening for colorectal cancer with a digital twin approach	The outcome of this study is over-diagnosis, which is not related to the patient's health.
No.2	Predicting transdermal fentanyl delivery using physics-based simulations for tailored therapy based on the age	The data of this study came from the team of Bahram et al., and the data was published repeatedly.
No.3	Retrospective study of glycemic variability, BMI, and blood pressure in diabetes patients in the Digital Twin Precision Treatment Program	The data of this study came from the team of Shamanna et al., and the data was published repeatedly.
No.4	Type 2 diabetes reversal with digital twin technology-enabled precision nutrition and staging of reversal: a retrospective cohort study	The data of this study came from the team of Shamanna et al., and the data was published repeatedly.
No.5	Reducing HbA1c in Type 2 Diabetes Using Digital Twin Technology-Enabled Precision Nutrition: A Retrospective Analysis	The data of this study came from the team of Shamanna et al., and the data was published repeatedly.

Supplementary Table 2. Retrieval strategy and results.

Source	No.	Search strategy	Results
PubMed	#1	"Patients"[Mesh]	84246
	#2	(Patient*[Title/Abstract]) OR (Client*[Title/Abstract])	8374723
	#3	#1 OR #2	8400972
	#4	Digital twin*[Title/Abstract]	1008
	#5	#3 AND #4	213
Embase	#1	'patient'/exp	3026190
	#2	patient*:ti,ab,kw OR client*:ti,ab,kw	12353537
	#3	#1 OR #2	12622850
	#4	'digital twin*':ti,ab,kw	991
	#5	# 3 AND #4	287
Cochrane library	#1	MeSH descriptor: [Patients] explode all trees	3827
	#2	(patient*):ti,ab,kw OR (client*):ti,ab,kw	1209580
	#3	#1 OR #2	1210142
	#4	(digital twin*):ti,ab,kw	69
	#5	#3 AND #4	40
CINAHL Plus with Full Text	#1	MH "Patients"	10931
	#2	SU patient* OR SU client*	781950
	#3	#1 OR #2	781950
	#4	SU digital twin*	146
	#5	# 3 AND # 4	14
Web of science	#1	patient* (Topic) or client* (Topic)	12004110
	#2	digital twin* (Topic)	13344
	#3	#1 AND #2	726
SinoMed	#1	"病人"[常用字段:智能] OR "患者"[常用字段:智能]	12931927
	#2	"数字孪生"[常用字段:智能]	95
	#3	#1 AND #2	6
CNKI	#1	(TKA% '病人' + '患者')	6321958
	#2	(TKA%= '数字孪生')	8042
	#3	#1 AND #2	18
WanFang	#1	主题:(病人) or 主题:(患者)	7776319
	#2	主题:(数字孪生)	6374
	#3	#1 AND #2	17
